# Performance Evaluation of Open Channel Buhlmann Fecal Calprotectin Turbo Assay on Abbott Alinity C Analyzer

**DOI:** 10.3390/diagnostics14161744

**Published:** 2024-08-11

**Authors:** Kavithalakshmi Sataranatarajan, Shishir Adhikari, Ngoc Nguyen, Madhusudhanan Narasimhan, Jyoti Balani, Alagarraju Muthukumar

**Affiliations:** 1 Department of Pathology, University of Texas Southwestern Medical Center, Dallas, TX 75235, USA; kavithalakshmi.sataranatarajan@utsouthwestern.edu (K.S.); madhusudhanan.narasimhan@utsouthwestern.edu (M.N.); jyoti.balani@utsouthwestern.edu (J.B.); 2 Core Laboratory, Clements University Hospital, University of Texas Southwestern Medical Center, Dallas, TX 75235, USA; shishir.adhikari@utsouthwestern.edu (S.A.); ngoc.nguyen@utsouthwestern.edu (N.N.)

**Keywords:** fecal calprotectin (fCAL), Buhlmann fCAL turbidometry assay on Abbott Alinity C analyzer (AFCAL), Buhlmann turbo assay, clinical performance

## Abstract

Inflammatory bowel disease (IBD) is characterized by chronic inflammation of the gastrointestinal (GI) tract. Fecal calprotectin (fCAL) is a noninvasive laboratory test used in the diagnosis and monitoring of IBDs such as Crohn’s disease and ulcerative colitis. The fCAL send-out test that our facility has been offering so far uses an ELISA-based method. In the current study, we sought to validate the performance of a Buhlmann fCAL turbo assay in an automated Abbott Alinity C analyzer (AFCAL) in our core laboratory. Five-day imprecision studies showed good performance for both within-run (5.3%) and between-day (2.5%) measurements. The reportable range was verified as 30–20,000 µg/g. Deming regression and Bland–Altman analysis indicated a strong correlation of r = 0.99 with a low, acceptable bias of 1.8% for AFCAL relative to the predicate Buhlmann fCAL ELISA results. AFCAL’s clinical performance was determined retrospectively in 62 patients with ICD codes for IBD. Overall, the implementation of AFCAL in our routine clinical testing has improved our turnaround time, reduced the cost per test, and significantly increased our clinician satisfaction.

## 1. Introduction

Chronic intestinal inflammation of the gastrointestinal tract is the hallmark of inflammatory bowel disease (IBD). Ulcerative colitis and Crohn’s disease are major forms of inflammatory bowel disease that are characterized by chronic recurrent episodes of inflammation in the gastrointestinal (GI) tract. Endoscopic procedures like colonoscopy, sigmoidoscopy, and capsule endoscopy are commonly performed to diagnose IBD [1]. Since the mild-to-severe form of this disease involves repeated examinations, performing these invasive means of evaluation can cause patient discomfort and result in unwarranted delays and become expensive. Along these lines, cost-effective, non-invasive tests have evolved to be valuable as there are little to no complications involved and they are less inconvenient for patients.

There are several serological markers to assess the severity of inflammation, like IL6, C-reactive protein (CRP), perinuclear antineutrophil cytoplasmatic antibodies (p-ANCA), lactoferrin, and lipocalin-2 (LCN2) [2,3,4,5,6]. Proteinase 3 antineutrophil cytoplasmatic antibodies (PR3-ANCA) have been shown to discriminate patients with ulcerative colitis from those with Crohn’s disease, along with demonstrating disease severity and activity [7]. Serum IL6 and sIL-2R, measured along with CRP and fecal calprotectin, have been shown to be a reliable marker to distinguish Crohn’s disease patients with intestinal inflammation [8]. Other serum biomarkers linked with IBD, such as Anti- Saccharomyces cerevisiae (ASCA-IgG, ASCA-IgA), bacterial flagellin (CBir1), and *Escherichia coli* outer membrane porin C (OmpC), are well documented [9,10,11,12]. Recently, fecal proteolytic activity and Oncostatin M (OSM), along with fecal calprotectin, have been shown to be reliable biomarkers of inflammatory bowel disease [13,14,15]. Calprotectin is a calcium- and zinc-binding protein of the S-100 protein family that accounts for 60% of the cytosolic protein in neutrophils. The suitability of calprotectin as a biomarker of choice for IBD is widely recognized due to the fact that it is released by neutrophils when they migrate to the affected area in the gastrointestinal tract during infection or inflammation [16,17]. Calprotectin can be measured in serum and in feces. While serum calprotectin reflects more systemic inflammation, fecal calprotectin (fCAL) is an indicator of local intestinal inflammation [18]. Elevation of fCAL is a sensitive measure of GI inflammation. Studies have shown that fecal samples provide better clinical sensitivity and/or specificity for IBD severity than serum [17]. In clinically quiescent ulcerative colitis, fecal calprotectin levels have been shown to strongly and reproducibly correlate with endoscopic and histological activity [19]. fCAL is not only used as biomarker in diagnosing irritable bowel disease but also to identify postoperative disease recurrence in IBD patients [20].

In 1992, Roseth et al. developed an enzyme-linked immunosorbent assay (ELISA) for calprotectin in feces (fCAL) as they found it to remain stable in feces up to 7 days at room temperature [16]. Nevertheless, the turnaround time was longer (~2 h) in the ELISA method and the samples had to be batch-tested. The Buhlmann fCAL turbo method (~10 min) was developed as an alternative to the ELISA method. The Buhlmann fCAL turbo method is based on a particle-enhanced turbidimetric immunoassay (PETIA) that uses antibodies covalently coupled to latex particles to quantify calprotectin in fecal samples with high precision and reproducibility in a fully automated process [21].

The initial FDA approval for the Buhlmann fCAL turbo assay was obtained for the Roche Cobas automated analytical platform. Recently, equivalency performance approval was obtained for a few other automated platforms, including Alinity C analyzers [22,23,24,25,26]. The Abbott Alinity C analyzer is one of the widely used automated chemistry analyzers in clinical laboratories and there are currently no field data available to independently verify the performance of this third-party Buhlmann fCAL turbo assay on the Alinity system. Therefore, the goal of the present study is to evaluate the analytical and clinical performance of the Buhlmann fCAL Turbo turbidimetric (fecal calprotectin) test as an open channel assay in the Abbott Alinity C analyzer (AFCAL) in our patient population.

## 2. Materials and Methods

**Figure 1:**
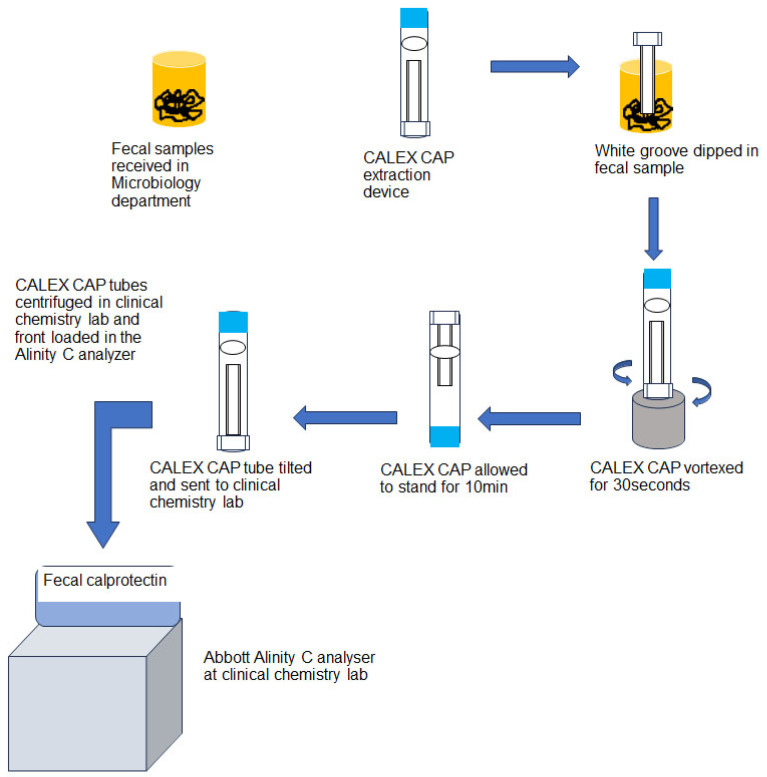
Flow chart of the fecal sample processing adopted from vendor kit insert.

This study was conducted at Clement’s University Hospital (CUH), UT Southwestern University, Dallas, USA. The institutional review board of the University of Texas Southwestern Medical Center approved this study. The evaluation of calprotectin turbo assay on Abbott Alinity C analyzer (Abbott Core Laboratory Systems, Lake Forest, IL, USA) was performed per the CLSI guidelines and manufacturer’s instructions. All experiments that involved clinical samples were performed using de-identified remnant (left over) samples as part of the in-house assay validation and performance evaluation. Fecal samples were processed per the manufacturer’s instruction (Buhlmann Diagnostics Corp., Amherst, NH, USA). The fecal samples received in our Microbiology department were processed using the CALEX CAP extraction device provided in the Buhlmann turbidimetry assay kit. The CALEX CAP tube has a white dosing tip with grooves at one end and a blue cap at the other. The white dosing tip was dipped into the stool 2–3 times at different positions to fill the grooves with stool sample completely. The tip was then removed and placed into the funnel of the CALEX CAP body and locked. The device with white head (dipping end) was vortexed for 30 s vigorously and allowed to stand for 10 min on the blue protection cap (dosing white tip on the top) ensuring that the stool was fully detached from the grooves. During this extraction process, the sample was diluted 500 times. The extracted fecal sample, now in the blue cap tube, was sent to the clinical chemistry lab for fCAL measurement. The fecal extracts were centrifuged and front-loaded on to the Alinity C analyzer. In the AFCAL assay, the fecal extracts were incubated with reagents and mixed with polystyrene nanoparticles coated with calprotectin-specific antibodies (immunoparticles). The calprotectin–immunoparticle complex formed was directly proportional to the fCAL concentration in the sample, which was measured by light absorbance (Buhlmann Diagnostics Corp., Amherst, NH, USA). The pictorial representation of fecal sample processing (Figure 1) was adopted from the vendor kit insert.

### 2.1. Precision Testing

We performed a 5-day imprecision study on the Abbott Alinity C analyzer to confirm the reproducibility within the runs and between different days. Two levels of quality control (QC) were tested in 4 replicates within one run and two runs were completed per day for a total period of 5 days. These data were used to estimate within-run, between-day, and total imprecisions.

### 2.2. Determination of Analytical Measurement Range (AMR) and Clinical Reportable Range (CRR)

To determine the AMR, we tested a set of 7 value-assigned samples provided by the manufacturer (Buhlmann Diagnostics Corp., Amherst, NH, USA). Each of the value assigned samples were run in triplicates on two different Alinity C instruments. CRR was further determined by 1:10 auto dilution of patient samples with values closer to the upper limit of AMR.

### 2.3. Method Comparison

We used *n* = 20 de-identified patient samples with known IBD clinical histories provided by the Buhlmann Diagnostics Corp. (Amherst, NH, USA). Buhlmann ELISA served as the predicate method for this comparison. Additional method comparison studies were conducted against the current sent out ELISA method performed by Mayo labs. For this qualitative comparison, we used the Buhlmann-recommended cut off ranges for the in-house AFCAL assay and the Mayo labs (ELISA) cut off ranges for the predicate method.

## 3. Data Analysis

All patient data were obtained from the University of Texas at Southwestern EPIC software system electronic records (UTSW EPIC) and de-identified for the analysis. Statistical analyses and graphs were generated using EP evaluator v12.2 (Data Innovations LLC, South Burlington, VT, USA) and Graphpad prism software v10.1 (GraphPad Software, La Jolla, CA, USA). Percentage coefficient of variation (%CV), scatter plot, Deming regression, and Bland–Altman plots were used to analyze the data.

## 4. Results

The precision study was conducted using two levels of QC materials provided in the kit. A total of 40 replicates were run over a period of 5 days for each level of QC. Both within-run and between-day imprecision for level 1 and level 2 QC were within the acceptable limit of 15%. Indeed, the intraday imprecision for levels 1 and 2 QC was 5.3% and 1.7%, respectively. The inter-day imprecision for levels 1 and 2 QC had a %CV of 1.9% and 2.5%, respectively (Table 1).

**Figure 2:**
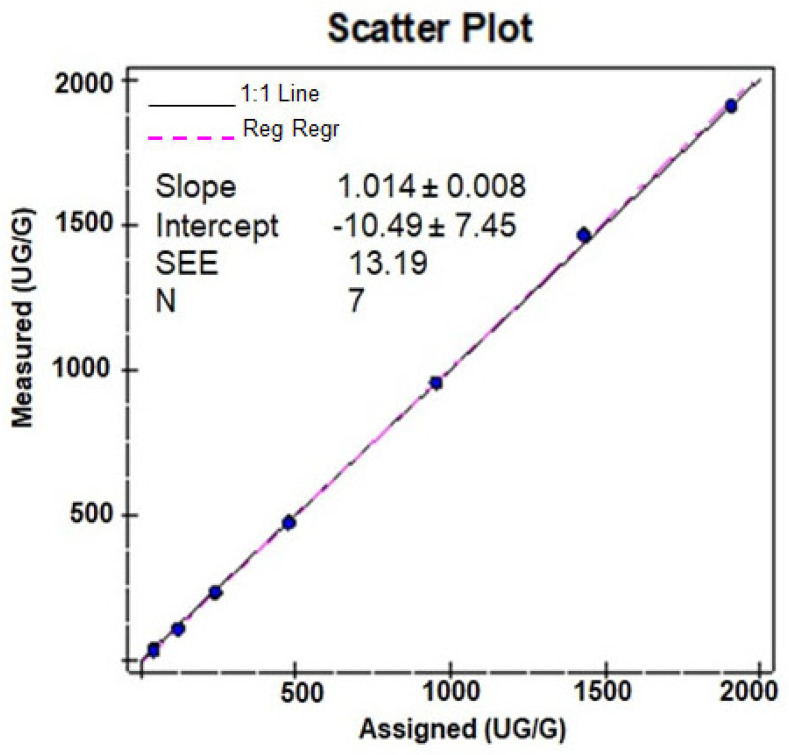
Scatter plot of the linearity study.

Linearity assessment was performed on two different Alinity C analyzers using seven levels of linearity materials in triplicate. In-house AFCAL assay showed a tight correlation and was found to be linearly proportional to the concentration of the mean of assigned and expected values (Table 2 and Figure 2). The mean recovery was 94.9% for all dilutions (with a minimum mean recovery of 78.1% and a maximum of 102.3% (Table 2)). The assay was linear over the tested AMR of 30–2000 µg/g. With 1:10 auto-dilution, the CRR was validated from 30 to 20,000 µg/g.

**Figure 3:**
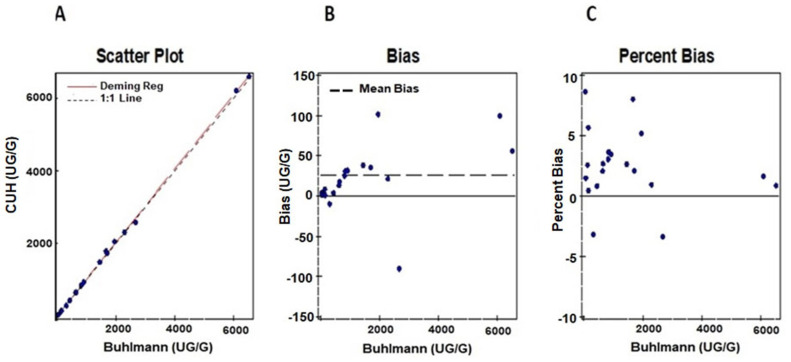
Comparison of AFCAL results to the predicate Buhlmann fCAL ELISA results shown in (**A**). Bias and percent Bias between the AFCAL and Buhlmann ELISA are shown in (**B**,**C**).

Method comparison studies were performed using *n* = 20 samples with known fCAL values provided by the vendor. Deming regression and Bland–Altmann plots for comparing in-house Buhlmann assay results against the predicate Buhlmann ELISA method are shown in Figure 3A–C. We observed a good correlation of r = 0.99 and an acceptable bias of 1.8% for the AFCAL assay compared to the predicate method.

We performed a Truth table qualitative comparison between AFCAL assay and our send-out (Mayo clinic laboratories) ELISA method using normal and borderline samples (*n* = 18). Manufacturer-recommended cut off ranges followed for AFCAL included ≤80 µg/g for normal, 80–160 µg/g for borderline, and ≥160 µg/g for a GI inflammation condition (abnormal). The cut off ranges used in the send-out ELISA method were <50 µg/g for normal, 50–120 µg/g for mild/borderline, and >120 µg/g for a GI inflammation condition (abnormal). Despite the noticeable differences in cut off levels between the two methods, there was a good qualitative correlation (84%) between them (Supplementary Table S1). Discrepancies were observed in three samples. One sample showed close agreement (within acceptable error limits) between the predicate and in-house methods, while the other two samples were totally discordant. 

**Figure 4:**
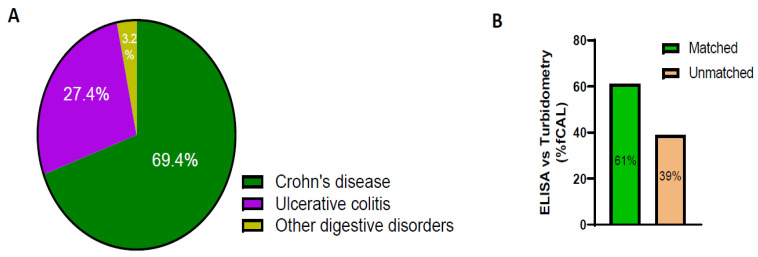
fCAL data comparison between ELISA and AFCAL assay. (**A**) Percentage of patients tested for ICD codes of Crohn’s disease, ulcerative colitis, and other digestive disorders. (**B**) Percentage of matched and unmatched fCAL values when comparing the fCAL values of the same patients in ELISA versus turbidimetry.

Next, we compared fCAL data from patient samples that had at least one fCAL value from the send-out ELISA method and one from the AFCAL method within a period of one year. Most tests were ordered for Crohn’s disease patients compared to those with ulcerative colitis or digestive disorders (Figure 4A). The AFCAL and ELISA methods yielded comparable qualitative results for 61% of the patient samples analyzed. However, for 39% of patient samples, the qualitative results did not match (Figure 4B).

**Figure 5:**
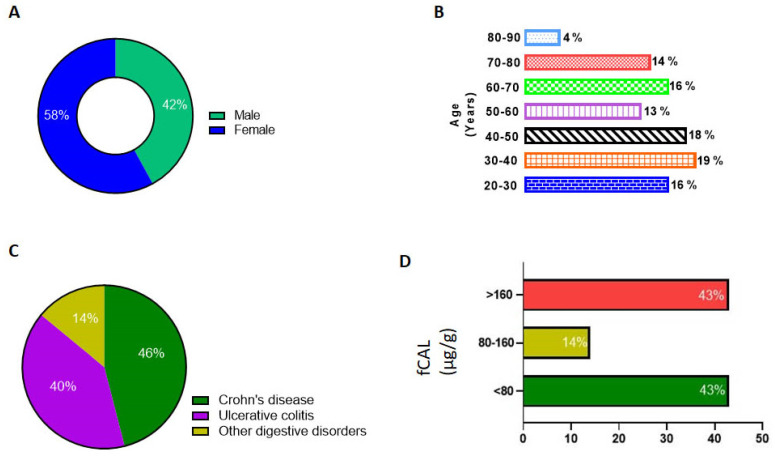
Data analysis of patient demographics and AFCAL results. (**A**) Percentage of male and female patients. (**B**) Age groups of patients tested for fCAL. (**C**) Percentage of patients tested for ICD codes for Crohn’s disease, ulcerative colitis, and other digestive disorders. (**D**) fCAL levels measured using Abbott Alinity C analyzer and percentage of patients in the normal, borderline, and abnormal fCAL level groups.

We then reviewed the fCAL levels in UT patients (*n* = 270) from the time the AFCAL assay was implemented in our clinical lab at UTSW. These tests were ordered for patients with ICD codes corresponding Crohn’s disease and ulcerative colitis. Only one latest fCAL value for each patient was considered for analysis. From the demographics data, we observed 58% of the patients were female and 42% were male (Figure 5A). A slightly higher percentage of fCAL was measured in age groups of 30–40 years (Figure 5B). As depicted in Figure 5C, around 45% of the fCAL orders were from patients with Crohn’s and ulcerative colitis ICD10 codes. We observed that 117 patient samples (43%) fell in the range of 30–80 µg/g. A total of 14% were in the mild/borderline inflammation range, i.e., between 80 and 160 µg/g, while 116 samples (43%) were above the cut off value, which is 160 µg/g. The individual fCAL values of these 116 samples ranged from 161 to 15,402 µg/g, suggesting an abnormal GI inflammation (Figure 5D).

We also performed experiments to ensure that there was no sample carryover. Lower level of QC for total bilirubin, albumin, calcium, and triglycerides were run in five replicates before and after running a high fCAL (>2000 µg/g). We observed a minimal carryover effect, and it was within the acceptability limit of 10%.

## 5. Discussion

The FDA 510k-cleared Buhlmann fCAL turbo assay has been validated on several analyzers [22,26], but not on the Abbott Alinity C analyzer, which is used in many diagnostic laboratories. This is the first study to verify the Buhlmann fCAL turbo assay on the Abbott Alinity C analyzer (Abbott Core Laboratory Systems, Lake Forest, IL, USA). The analytical performance of the AFCAL assay measured in terms of precision and linearity was within the acceptable limits (15%). Sample carryover was also minimal and within the expected limits.

The Buhlmann fCAL turbidimetry assay has been validated in a few other platforms like Vitros 5600 analyzer [22,25], Optilite benchtop analyzer [26], Mindray BS-200E, and Cobas-c111 [24]. The analytical performance of our new in-house AFCAL assay is comparable with that of other automated analyzers. A notable aspect of the Buhlmann turbidimetry assay is its quick result time of approximately 10 min, compared to about 35 min for Diasorin and nearly 2 h for the Buhlmann ELISA. More importantly, the CALEX CAP method for extraction from the Buhlmann turbidimetry assay simplifies the process compared to the older weighing methods. This critical advancement significantly reduces processing time by eliminating the need for weighing and buffer addition during extraction.

In our study, there was a strong correlation between the AFCAL assay and the Buhlmann ELISA. However, the comparison between our in-house assay against the send-out referral lab method showed varying levels of agreement. The good correlation with the Buhlmann ELISA method may be attributed to the use of the same antibodies in both assays. It is important to note that fCAL assays are not standardized across different vendor platforms [22,23,24,25,26,27,28]. This could explain the discrepancy between our AFCAL assay and the send-out methods, alongside differences in antibodies and variations in assay design. We adhered to the manufacturer’s cut off values for the AFCAL assay: normal ≤80 µg/g, borderline 80–160 µg/g, and abnormal ≥160 µg/g. In contrast, the send-out lab uses different cut off values: normal <50 µg/g, borderline 50–120 µg/g, and abnormal >120 µg/g (https://www.mayocliniclabs.com/test-catalog/overview/63016#Clinical-and-Interpretive) accessed on 5 August 2024. Due to the differing reference values between the two assays, we only conducted a qualitative comparison using normal, borderline, and abnormal results. Discrepancies in reports may also stem from differences in sample extraction procedures, variation in patients’ drug treatment regimens, and differences in the timing of sample collection and measurement between the methods. The analysis of patient data revealed variability in fCAL levels at different time points for the same individual. Some patients’ levels returned to normal levels within a few months, while others exhibited increasing levels. This variability may be due to differences in treatment regimens, lifestyle changes, and medications that patients are using at the time of the blood testing. Further investigation is needed to explore these factors, which are beyond the scope of the present study.

fCAL is a biomarker for mucosal inflammation, and its level changes in response to treatment, relapse, and post-operation. Besides its significance in ulcerative colitis and Crohn’s disease, elevated fCAL levels have also been linked to colorectal cancers [29]. Due to its simple stool-processing method and shorter turnaround time, Buhlmann turbidometry is routinely ordered to detect inflammation in the GI tract. Recent findings have validated the performance of the fCAL-turbo assay, showing a strong correlation with endoscopic activity in IBD patients [30]. While analyzing patient data, we observed that the AFCAL assay was performed multiple times within a year for some patients, often at short time intervals, without the use of invasive procedures like colonoscopy and endoscopy. By utilizing fCAL measurements over time, longitudinal fCAL profiles can be created, allowing for appropriate management of GI inflammation. Our patient data clearly indicates that transitioning from the send-out ELISA method to the in-house Buhlmann turbidometry assay on the Abbott Alinity C analyzer (AFCAL assay) has been advantageous. The use of the AFCAL assay has notably reduced turnaround time for patient results, lowered the cost per test, and greatly improved satisfaction among both clinicians and patients.

## 6. Conclusions

Our study is the first to demonstrate the use of Abbott Alinity C analyzer for measuring fCAL using the particle-enhanced Buhlmann turbidometry assay. The analytical and clinical performance of this assay in Abbott Alinity C analyzer makes it an efficient tool for fCAL measurement in clinical laboratories, thereby reducing costs and turnaround times for patients.

## Figures and Tables

**Table 1:** Precision of Buhlmann turbo method in Abbott Alinity C analyzer (AFCAL).

	Within-Day (*n* = 40)		Between-Day (*n* = 40)	
QC	Mean (µg/g)	%CV	Mean (µg/g)	%CV
QC Level 1	85	5.3	84	2.5
QC Level 2	291	1.7	290	1.9

**Table 2:** Linearity assessment of AFCAL assay.

	N	Est	Mean	Residual	% Recovery
39.7	3	29.76	31.00	1.24	78.1
119.2	3	110.35	105.43	−4.91	88.5
238.4	3	231.18	229.83	−1.35	96.4
476.9	3	472.95	472.37	−0.58	99.0
953.5	3	956.08	954.13	−1.94	100.1
1430.6	3	1439.71	1463.60	23.89	102.3
1907.5	3	1923.15	1906.80	−16.35	100.0

## Data Availability

The data are not publicly available due to ethical reasons. However, upon request, the data presented in this study can be shared.

## Supplementary Materials

The following supporting information can be downloaded at: https://www.mdpi.com/article/10.3390/diagnostics14161744/s1, Table S1: Qualitative comparison of Alinity fCal results (in house, UTSW) against our referral ELISA test.
