# Supplementary material for: Performance Evaluation of Open Channel Buhlmann Fecal Calprotectin Turbo Assay on Abbott Alinity C Analyzer
Source: Diagnostics (Basel). 2024 Aug 11;14(16):1744. doi: 10.3390/diagnostics14161744 (PMC11353904; doi:10.3390/diagnostics14161744)
Supplement: Supplementary file 1 [file diagnostics-14-01744-s001.zip › diagnostics-3109032-supplementary.pdf]

Supplementary Table S1

Sample ID	Mayo Result	Result	UTSW Result	Result
Normal	<50 µg/g		<80 µg/g	
1	<50	NORMAL	21.4	NORMAL
2	<50	NORMAL	0	NORMAL
3	<50	NORMAL	15.1	NORMAL
4	<50	NORMAL	64.2	NORMAL
5	<50	NORMAL	35.7	NORMAL
6	<50	NORMAL	74.1	NORMAL
7	<50	NORMAL	3.2	NORMAL
8	<50	NORMAL	0	NORMAL
9	<50	NORMAL	14.4	NORMAL
10	<50	NORMAL	0	NORMAL
11	<50	NORMAL	10.2	NORMAL
12	<50	NORMAL	8.8	NORMAL
13	<50	NORMAL	0.4	NORMAL
14	<50	NORMAL	68	NORMAL
BORDERLINE	50-120 µg/g		80-160 µg/g	
1	53.9	BORDERLINE	88.8	BORDERLINE
2	57.5	BORDERLINE	68	NORMAL
3	66.3	BORDERLINE	1094	ABNORMAL
4	69.1	BORDERLINE	198.1	ABNORMAL

Supplemental Table S1. Qualitative comparison of Alinity fCal results (in house, UTSW) against our referral ELISA test.
